# “It's My Dark Secret”: A Qualitative Study on the Abortion Experiences of US Active‐Duty Servicewomen

**DOI:** 10.1111/psrh.70064

**Published:** 2026-04-29

**Authors:** Caitlin Russell, Tiara Walz, Laura Manzo, Shelby Mueller, Sharon Messina, Sharon Arana, Keira Feng, Holly Harner

**Affiliations:** ^1^ University of Pennsylvania Philadelphia Pennsylvania USA; ^2^ US Army Medical Center of Excellence San Antonio Texas USA; ^3^ Yale University New Haven Connecticut USA; ^4^ University of Massachusetts – Boston Boston Massachusetts USA; ^5^ US Air Force Washington D.C. USA; ^6^ Jefferson College of Nursing Philadelphia Pennsylvania USA

## Abstract

**Introduction:**

The Hyde Amendment limits US military abortion care and coverage for active‐duty servicewomen (ADSW). ADSW face numerous barriers to care when seeking an abortion. The purpose of this study was to explore the lived experiences of US servicewomen who had an abortion while serving on active duty.

**Methods:**

Researchers conducted a qualitative secondary analysis of free‐text response data using thematic analysis. We developed the codebook using a modified version of Coast et al.'s (2018) framework for *Trajectories of women's abortion related care.* Data were collected between December 2021 to June 2022 and included *N* = 178 participants who self‐reported having an abortion while serving on active duty in the US military.

**Results:**

Most participants (*n* = 122, 62%) cited unplanned pregnancy as their reason for having an abortion. Some participants (*n* = 22, 12%), identified as having a “medically necessary” abortion (i.e., miscarriage, ectopic pregnancy). Emergent themes included lack of understanding about abortion care, financial and logistical barriers to accessing abortion care often exacerbated by military policies, lack of medical privacy, and subpar care received from military treatment facilities during pregnancy options counseling or TRICARE covered abortion care.

**Discussion:**

ADSW experience systemic barriers to care and often seek care outside of the military medical system to protect their privacy. Military healthcare professionals do not provide adequate pregnancy options counseling or patient education regarding pregnancy loss. The US Department of Defense should enact policies ensuring access to abortion care to maintain the physical well‐being and mission readiness of servicewomen.

## 1 Introduction

As of 2024, women make up 17.9% of United States (US) active‐duty servicemembers with nearly 227,000 servicewomen serving across the Army, Navy, Air Force, Space Force, and Marines [1]. Almost 95% are of reproductive age (18–45 years‐old) with the majority being 20–29 years old [2]. US servicewomen report systemic barriers to contraception access and have higher rates of unintended pregnancies when compared to civilian women [3]. Women between the ages of 20–29 account for over half of induced abortions in the US, and up to 60% of unintended pregnancies are ended with an induced abortion, indicating that US servicewomen have predisposing factors that increase their likelihood of needing an abortion at some point during their lives [4, 5].

Although the US Department of Defense (DOD) does not track the number of abortions obtained by active‐duty servicewomen (ADSW), it is estimated that 2500–4100 have an abortion annually [6]. These RAND estimates are based on inferences from self‐reported data in the 2020 DOD Women's Reproductive Health Survey, however, due to stigmatization associated with obtaining abortion care and high levels reporting bias which often impact abortion research, these numbers likely underestimate the true scope of this vital reproductive healthcare [6, 7]. For the purposes of this paper, we use Kumar et al.'s (2009) definition that abortion‐related stigma is “a negative attribute ascribed to women who seek to terminate a pregnancy that marks them, internally or externally, as inferior to the ideals of womanhood” (p. 628) and has been found to impact ADSW in terms of personal and professional repercussions stemming from their decision to have an abortion [8, 9]. Due to the nature of military regulations which limit freedom of travel and changing jobs or units, ADSW can experience more severe repercussions from abortion stigma compared to civilian women such as professional retaliation, fear of being reported by their medical providers and inability to access abortion care or post‐abortion care resources [10].

The Hyde Amendment prohibits federal funding from being used to pay for abortion care except in cases of rape, incest or when the mother's life is endangered [11]. TRICARE, the mandatory healthcare program for servicemembers, is funded annually by the Department of Defense Appropriations Act and limits abortion coverage to cases of rape, incest, or if the mother has a life‐endangering condition [12, 13]. TRICARE designates abortions meeting Hyde Amendment criteria as “covered” and will pay associated costs, and all other abortions as “non‐covered” [12, 14]. Under these provisions, ADSW are not permitted to pay out of pocket for abortion care at Military Treatment Facilities (MTFs) [12]. ADSW seeking “non‐covered” abortion care must pay out of pocket for all costs associated with “non‐covered” abortion care and seek care at non‐MTF clinics [15]. Abortion care is unique in this aspect, as no other health condition or nonelective medical procedure requires servicemembers to seek out their own providers without input or guidance from military healthcare professionals and to pay in full for all travel and costs associated with the procedure. While on active‐duty, servicemembers are provided all other care at no‐charge by medical professionals at their home station or compensated for travel costs if they need to be sent to another MTF or civilian facility for specialized care (e.g., cancer, surgery) [16]. Accessing care for any other health condition is as simple as attending “sick call” which is offered daily or making an appointment with one's primary care provider [17, 18].

Previous research indicates between 66% and 78% of servicewomen seeking abortion care felt they could not have a child at the time of pregnancy [19, 20]. Many servicewomen experience systemic barriers to accessing reliable contraception, resulting in unplanned pregnancies that lead.

ADSW to pursue abortion care [21]. Barriers to abortion care include logistical barriers (e.g., stationed overseas, financial burden), legal barriers (e.g., host nation abortion bans, state level abortion bans), and institutional barriers (e.g., fear of personal or professional repercussions by leadership for having an abortion) [19, 20]. Logistic and institutional barriers are compounded by DOD regulations requiring servicemembers to request permission and leadership approval for time off or travel away from their military post during nonduty hours (for any reason) [22]. These systemic barriers seemed to undermine “mission readiness,” the concept that servicemembers have the ability to execute their duties anywhere at any time, by eroding trust in unit cohesion and degrading their overall health, physical fitness, and financial well‐being [23].

Despite these challenges, little research exists on the experiences of ADSW seeking access to abortion care [24]. This study seeks to address existing gaps in knowledge by exploring the lived experiences of servicewomen who had an abortion while serving on active duty in the US military.

## 2 Methods

### 2.1 Design and Recruitment

This qualitative study is a secondary analysis of free‐text response data conducted using thematic analysis [12]. A military community organization engaged in a collaboration with academic partners to design a questionnaire to gather data to inform policy makers about reproductive healthcare policy knowledge and access among ADSW. Convenience and snowball sampling was performed by disseminating recruitment emails to members of an organization whose membership required validation of active‐duty status. Our sample (*N* = 178) is a subset of a larger dataset (*N* = 1124) which explored the policy knowledge and the lived experiences of servicewomen seeking reproductive healthcare. Inclusion criteria for the secondary analysis were: (1) currently on active‐duty; (2) completed the questionnaire; and (3) self‐reported having an abortion while serving on active‐duty. This study was granted exemption status by the Institutional Review Board.

### 2.2 Data Collection and Analysis

A military community organization collected data between December 2021 to June 2022. To maximize anonymity and encourage participation, they did not collect demographic data. Participants responded to a 24‐item questionnaire which included yes/no questions and free‐text responses (see Data S1). Questions explored the lived experiences of servicewomen on active duty receiving reproductive healthcare and assessed their knowledge of sexual and reproductive health policies. Participants had to answer all dichotomous questions to submit the survey. For free‐text questions, response rates ranged from 33% to 90% and varied in length from one word to several paragraphs with the longest average responses addressing experiences of discrimination (average of 34 words) and general reflections (average of 58 words), and the shortest describing financial difficulty (average of six words) due to the number of “yes” or “no” responses (see Data S2).

The military community organization then collaborated with this research team to perform a secondary analysis of the existing data. The research team generated descriptive statistics for a sample characteristic question (Question 5, see Data S1) asking participants why they sought abortion care (see Table 1). We developed the codebook using a modified version of Coast et al.'s (2018) framework for *Trajectories of women's abortion related care* [25]. We selected this framework a priori due to its focus on the individual and the time‐sensitivity associated with accessing abortion care. Modifications included the addition of military‐specific considerations (e.g., military regulations, Uniform Code of Military Justice, MTF versus civilian facilities) to better capture unique factors that impact servicewomen seeking abortion care. We used MAXQDA 2024 to organize data and thematic analysis methods to analyze qualitative data [26, 27].

**TABLE 1:** Participants' primary reasons for having an abortion.

Primary reason for abortion	
Rape	11 (6%)
Incest	0 (0%)
Danger to your health	18 (10%)
Concerns about health of baby	19 (11%)
Unplanned pregnancy	111 (62%)
Other	15 (9%)
Did not answer	4 (2%)

Three researchers trained in qualitative methods independently coded data, and met on a weekly basis to discuss results, resolve discrepancies, mitigate bias, and document changes to ensure rigor and reflexivity (see Data S2) [26].

## 3 Results

We organized the results in accordance with Coast et al.'s framework which comprises three main domains Individual Context (e.g., Knowledge and Beliefs about Abortion; Individual Characteristics), Abortion‐Specific Experiences (e.g., Disclosure and Non‐Disclosure; Access to Abortion Provider), and International/Sub‐National Context (e.g., Military Legal and Regulatory Environment; Military Health System) (see Table 2) [25].

**TABLE 2:** Exemplar quotes.

Domain: Individual context	
Knowledge and beliefs about abortion	“Abortion care is healthcare.” “I knew I didn't want to have the child and was in a state where I had access to termination clinic with the procedure. Since being on [active duty], I have had 2 children and am currently pregnant with a 3rd. I do not regret my decision to prioritize myself and starting my career at 21 over becoming a parent.” “I remember not knowing who or what my resources were, so I just went into the community. Fortunately, Texas at the time allowed abortions. I had one and my now husband and I are grateful for the opportunity to have access to safe healthcare that allowed us to continue our careers and life on our terms.”
Individual characteristics	“I did not have the funds and had to borrow money.” “I was 23 and newly engaged when I found out I was pregnant. However, my now‐husband and I had just commissioned and had no chance of being stationed together for at least 2–3 years. I was not ready to have a baby, especially on my own.” “I did not want the military to know [I was getting an abortion], and I did everything on my own dime over a weekend, so I had to travel to a location with facilities that could perform it.”
Domain: abortion‐specific experiences	
Disclosure and nondisclosure	“The only people I told about this pregnancy were my partner, my commander and my Primary Care Manager. As for leadership‐ commanders need to be very delicate in who knows about pregnancy of member‐ especially if there will be a termination. My leadership team knew I was pregnant, including first shirt [senior ranking sergeant]. But only person I told about plans to terminate was commander. This made it awkward for me because first shirt was confused when I was no longer pregnant and actually made me feel judged. As an officer‐ I really only wanted to share this personal decision with my commander.” “Somehow my work center found out about my abortion. Airmen gossiping as usual. I was called a murderer, treated unfairly, and ostracized. I was absolutely miserable and wanted to unalive myself.” “I had to seek access to have an abortion outside of the military because it was not covered by Tricare, and at the time would have to seek my commander's approval. I decided to terminate the pregnancy, because my personal choices of my body are my business, not anyone else's and did not get approval from my MALE very RELIGIOUS commander.”
Access to abortion provider	“My experience was when I was Navy enlisted and overseas and [the abortion] was not provided by a US military or civilian provider, rather a foreign national conducted the procedure.” “Because of the location I was stationed (rural) I had to drive 4 h to a city to receive treatment.” “I was lucky enough that the state I was in had a women's clinic so I was able to get it on my own terms and no one knowing that I didn't want to.”
Domain: International/sub‐national context	
Military legal and regulatory environment	“I was not given the same aftercare in women's health that is given for a miscarriage. I was not offered any convalescent leave.” “With severe restrictions about to be emplaced across vast states in the South and Midwest [on abortion access], women should be compensated for the fact that services can no longer be performed remotely close to duty locations.” “I had to address my leadership and beg to have my PT test pushed back even though keeping the pregnancy would have resulted in no test at all.”
Military health system	“Military OB told me about a local source but they were unable to take me. Had to google a clinic and it was over an hour away.” “I was judged by base clinic personnel for making the decision to abort. After taking a pregnancy test at the clinic (2014), receiving the news that I was indeed pregnant, and telling the staff there that I fully intended to terminate the pregnancy; I had to fight with the staff to prevent them from loading a pregnancy profile in the system for me. I did not have a good relationship with my CC and I did not want him knowing I was pregnant out of wedlock.” “The OB clinic at [military base] marked me as a no show after I had my first appointment and did not help any further after the termination. They told me that I had to follow up with my [Primary Care Manager] even though I had an open referral with their office. They would not help with convalescent leave after the procedure and kept telling me I had to get commanders approval before they would process convalescent leave. They would not answer any questions about what to expect, or if I had to bring in any paperwork for medication or procedure after.”

### 3.1 Domain One: Individual Context

Coast et al. (2018) define the individual context as “characteristics that influence whether a woman obtains abortion‐related care” [13, p. 201], including factors such as morality views on abortion and individual characteristics. This domain includes the themes Knowledge and Beliefs about Abortion and Individual Characteristics.

#### 3.1.1 Knowledge and Beliefs About Abortion

The majority of participants (*n* = 122, 62%) cited an unplanned pregnancy as their primary reason for termination (see Table 1). These participants viewed abortion as a means to achieve a personal or professional goal, such as continuing their military career or attaining their personal fertility goals. One participant stated that it “Wasn't the right time. Had extended training coming up, and a pregnancy would have set me back, potentially removed me from training.” Another stated, “I didn't and don't want kids.”

Sample participants (*N* = 178) who identified as having a “medically necessary” abortion (i.e., threat to health, fetal anomaly) in the free‐text response section (*n* = 22, 12%) felt their situation was different from those who had an induced abortion for personal or professional reasons. One participant stated, “termination of a wanted pregnancy is not the same as a ‘typical’ abortion and should not be treated in the same manner”. Several felt that because the termination was medically necessary, the DOD should offer more support through mental health counseling, financial coverage, and convalescent leave. Of these 22 participants, 18 self‐reported ectopic pregnancies or cases of medical miscarriage management (the use of abortifacients or a dilation and curettage to remove retained tissue from a fetus with no cardiac activity). These responses reveal the difficulties associated with the language used surrounding abortion care and the possibility of provider‐patient education deficits. Although research highlights inconsistencies in how nonmedical personnel define medical miscarriage management in relation to abortion care, clinicians should ensure that patients understand what constitutes a missed miscarriage [28, 29]. Participants' responses potentially reveal a lack of understanding regarding the care they received, as these treatments do not clinically constitute the “termination” of a viable pregnancy.

My body never recognized the miscarriage. I waited 2 weeks before deciding to take the abortive, hormonal drugs. I still wonder if I killed my baby or if the miscarriage was real.

#### 3.1.2 Individual Characteristics

This subtheme is defined by participants' partner/family/community context as well as their sense of self‐efficacy/agency/autonomy/power and how these factors influence an individual's ability to obtain abortion care [25]. Participants frequently highlighted these influences in terms of paying for their care or the necessity of traveling more than an hour to access services. Those with supportive friends and family described how “my friends crowdfunded for me” or “my partner assisted in covering costs” so they could obtain abortion care. However, participants acknowledged disparities in support, noting that while some had “help from the father of the baby, not everyone has that.” Participants who felt unsupported often described more challenging circumstances, including situations involving “danger to life of me and baby (abuse)” or otherwise were in “a relationship that was not healthy.” These individuals were more likely to report experiencing financial difficulty affording an abortion with costs ranging from a few hundred to a few thousand dollars.

In a military setting, self‐efficacy is particularly complex due to the strict travel and leave regulations that servicewomen must follow, which have no civilian sector equivalent. One participant stated, “I was a young Captain in an unhealthy relationship that feared having my entire command notified. I took a pass and drove [out of state].” Even when participants successfully accessed abortion care, their self‐efficacy and autonomy was often limited by unit requirements as they were expected to continue performing their duties:

Technically I wasn't authorized leave [time off approved by a military superior]. I asked for leave but was denied due to being on shift work. It was an uncomfortable process—my supervisor denied the 24‐h quarters request, so I called the on‐call [military] nurse to get a day to rest through the cramps and gather myself. I'm a public facing figure at work. That gave me until the next morning to figure everything out and recover a little. I was afraid of bleeding through on shift—[the abortion clinic] wouldn't let me leave with the [abortion] pill and take it on the weekend when they were closed. I had to take it onsite before going back to work.

### 3.2 Domain Two: Abortion‐Specific Experiences

Coast et al. (2018) define abortion‐specific experiences as the actions taken by women attempting to access abortion care that are influenced by their personal circumstances and factors within their broader macro‐environment. This domain includes themes about participants' ability to disclose or not to disclose their abortion care and their ability or inability to access an abortion provider.

#### 3.2.1 Disclosure and Non‐Disclosure

Most participants who knew they would terminate their pregnancies avoided notifying their leadership due to fears of personal and professional repercussions, with several reporting they told no one and one participant adding “It's my dark secret.” One participant stated, “I only told a few select friends, no leadership because I was scared of how I would be treated.” Others felt unable to share their decision with anyone due to the pervasive stigma of abortion in the military. One participant explained, “When I sought abortion I went around the military system because of the stigma at work, especially since at the time I thought I would have to get my [chain of command]'s permission for an ‘elective surgery’. That is what was explained to me before.”

The ability to discreetly access abortion care was critical for servicewomen seeking to maintain their privacy. One participant noted, “I was lucky enough that the state I was in had a women's clinic so I was able to get [an abortion] on my own terms and no one knowing that I didn't want to.” Others “had to make up an excuse for taking leave—as an FGO [Field Grade Officer] I was uncomfortable letting supervisor know the real reason.”

Some participants were forced to disclose their intent to seek abortion care due to military regulations requiring permission to travel or exemptions from mandatory physical training. One participant stationed overseas required authorization to travel and “had to get permission from my flight commander and squadron commander. It was embarrassing having to explain myself each time.” Another participant had to “address my leadership and beg to have my [mandatory] PT test pushed back,” so she could seek care and physically recover following her abortion.

MTFs, at times, failed to ensure patient privacy with some servicewomen having to publicly disclose their abortion care needs. One participant was forced to “announce [my] termination at the front desk when I asked if I could update them not in the lounge” instead of being provided a private space. Even after obtaining abortion care, participants expressed concerns about disclosing their decision to military healthcare professionals during future appointments, fearing judgment.

To maintain unit readiness, military leaders receive reports on their personnel with medical conditions that render them nondeployable. Each of these conditions is identified by a discrete assignment limitation code. Because pregnancy is a nondeployable condition, participants who terminated were concerned that reports noting their prior pregnancy would not be updated to reflect their recent termination, thus showing them as nondeployable.

The emotional turmoil was also extended by the process of removing my assignment limitation code and profile by Public Health […] I was desperate to get the code removed before my [unit medical report] was pulled […] anyone that noticed it in my chain of command would be likely to comment on [the pregnancy] in a congratulatory tone and I would have lost it.

#### 3.2.2 Access to Abortion Provider

The first step for servicemembers to access healthcare is to schedule an appointment with a military healthcare provider. However, because the Hyde Amendment prevents TRICARE from covering most abortion services, and because MTFs are not permitted to perform abortion care for “non‐covered” abortions, even those participants who had empathetic encounters with military healthcare professionals when their pregnancies were confirmed were unable to access abortion care through these channels: “The providers were very professional and empathetic but had their hands tied for abortion services.” Participants assigned to providers with anti‐abortion beliefs faced additional hurdles, needing to make additional appointments to access care, such as one who reported, “My PCM [Primary Care Manager] didn't agree with abortions, so I had to find a different [MTF] provider to initiate the process.”

Delays in accessing abortion care were further exacerbated by MTF protocols which dictated prolonged timelines for pregnancy confirmation. One participant shared, “Where I am stationed they won't even see you until eight weeks, and you don't even get an ultrasound until 12—which 12 weeks would be late for any potential termination.” Additionally, participants reported scheduling challenges when trying to make an appointment with nonmilitary abortion providers, noting many were “not available on weekends or federal holidays,” times when it was easier to get permission from leadership to take time off from work for travel.

ADSW stationed overseas experienced additional obstacles that delayed access to care, such as international travel over significant distances like “Seoul to North Carolina.” Those able to access care in foreign countries encountered language barriers that further delayed treatment and often required additional travel: “I had to find a clinic who could speak English or allow a translator. The closest facility would not take me because I didn't speak the language and they didn't allow translators into a procedure room.”

Many questioned the quality of care they received overseas, and felt that DOD policies did not facilitate best practices for ensuring the health of servicewomen:

I terminated a pregnancy for medical reasons and had to go to a clinic in a foreign country. I ended up getting sepsis due to unsanitary conditions, paying $5000.00 USD, and ended up back at the MTF for an abortion because of the sepsis. Had Tricare offered a safe abortion in the first place, I would not have undergone the traumatic events I did. Furthermore, the events of my septic abortion led to complications of future pregnancies, costing Tricare much more money (C‐sections) in the long haul.

### 3.3 Domain Three: International/Sub‐National Context

Coast et al. (2018) define this domain as the structural and institutional frameworks (e.g., legal setting, health care systems) in which a woman seeks to obtain an abortion, extending from the individual's immediate community to the broader international context. This domain includes the themes of the Military Legal and Regulatory Environment and the Military Health System.

#### 3.3.1 Military Legal and Regulatory Environment

The Hyde Amendment remains the most influential regulation impacting servicewomen's access to abortion care as the Department of Defense Appropriations Act, which funds TRICARE, cannot pay for abortion care unrelated to rape, incest, or if the mother's life is endangered. TRICARE does not cover abortion for severe fetal abnormalities. Four participants obtained abortion care later in their pregnancies due to fetal abnormalities such as anencephaly. One described her experience when

During my first pregnancy we found out at 21 weeks during our anatomy scan that our fetus had anencephaly. This is a condition that is not compatible with life as the brain is not fully developed‚ All with the condition die and it often creates complications for the mother but is not classified by Tricare as dangerous to the mother. We found out while talking to the health care providers after the scan that the military would only provide continued health care if I carried to term.

Despite the severity of these conditions, these servicewomen had to pay for their abortion care entirely out‐of‐pocket due to TRICARE restrictions. Participants felt frustrated with this policy as they considered their pregnancies to be a risk to their health and future fertility. One found it “tough to understand why these entities wanted me to carry a baby full term that was brain‐dead and not viable.”

Additionally, the medical conditions that qualify as endangering the life of the mother under TRICARE are limited. Conditions that do not meet the restrictive criteria can still pose significant risks to the mother's health and jeopardize future fertility plans.

I had premature rupture of membranes at 16 weeks. I was not in labor. I was told my baby couldn't survive in my uterus with no amniotic fluid and there was a risk of me not being able to have more children if I developed an infection […] I was told if I chose to terminate pregnancy, which was the recommendation, I would have to pay out of pocket because TRICARE wouldn't pay for it unless I was in active labor or if I had an infection. I wasn't in active labor and I didn't have an infection. My doc told me that every day the baby remains in my uterus with no fluid I put myself at risk of developing an infection which could result in a hysterectomy. I opted to terminate the pregnancy.

Several participants reported being counseled that TRICARE would not cover any complications resulting from a “non‐covered” abortion. One participant stated, “[My PCM] ensured I understood the consequences of any negative effects from the termination regarding TRICARE's lack of coverage for any resulting issues due to elective procedures.” Others expressed their frustration that no other medical conditions imposed restrictive coverage limitations on servicemembers. Participants also voiced concerns regarding barriers to care related to leave and pass policies, noting this as an area where the DOD could intervene: “I understand that it's difficult for TRICARE to pay for abortions, but providing medical leaves and support for members to access that option is taking care of the military member in a time of need.”

The military job environment further negatively impacted access to abortion care as some participants reported that leadership cited personnel's professional duties to deny or limit leave and pass. Many participants described arguing for specific days off to meet abortion clinic appointment requirements, while striving to minimize their time away from their jobs. One participant “did it on a weekend so I could be back for my shift Monday night working Ops” and another who did not want her abortion to “interfere with my duty day […] was able to elect when to induce evacuation, and not miss any duty days.” Yet another participant, instead of taking time to physically recover, “took a PT test and went to the range out of fear of discrimination if I told anyone,” following her abortion.

For health and safety reasons, certain military roles are not open to pregnant servicewomen (e.g., positions requiring deployment, hazardous material exposure etc.). However, abortion care was sometimes used to justify barring ADSW from critical career development opportunities and roles due to these regulations, even though they terminated their pregnancies. One participant was “told I couldn't apply for specific jobs within a set amount of time after the abortion despite all follow up appointments and checks being done well before that time.”

#### 3.3.2 Military Health System

Although the MTFs rarely provide direct abortion care, even if a servicewoman qualifies for a “covered” abortion, they are often the first point of contact for servicewomen seeking to confirm their pregnancy and explore their options. Although military healthcare professionals are not prohibited from performing pregnancy options counseling, some participants found military providers were unable to provide them with information or resources to obtain abortion care. One participant bemoaned the “lack of common healthcare provider (OB and primary) awareness of policies for advising and helping members access abortions” and another stated “It's up to the person to figure it out on their own, especially in a foreign country.” In some cases, clinic staff seemed unwilling to assist servicewomen who sought abortion care, with one participant describing how the OB clinic:

Marked me as a no show after I had my first appointment and did not help any further after the termination. They told me that I had to follow up with my [primary care provider] even though I had an open referral with their office. They would not help with con leave […] they would not answer any of my questions about what to expect.

The Military Health System permits medical personnel to abstain from providing care, such as contraception or abortion care, if it conflicts with their personal beliefs. However, a few participants found that their healthcare providers were knowledgeable about abortion‐related policies and willing to support their decision:

My PCM [Primary Care Manager] went above and beyond to help me navigate the policies for seeking an elective termination […] She held my hand through making sure what I was entitled to in terms of a fitness profile and record of my termination were captured in my record.

Among the few participants who had received abortion care through MTFs, there was still a prevailing sense of judgment and inadequate communication among healthcare staff regarding the reason for hospitalization.

I felt discriminated against because when I was finally admitted to the Naval Hospital and they had HHQ permission to perform the abortion, I was placed at the very end of the hall, told that a certain nurse did not want to be a part of my case and, after the fact, no care was taken to inform others that I had had an abortion. The lactation consultant came in my room the next day and asked, “Where's the baby?” While receiving my medicine from the pharmacy, the clerk asked if I was breast feeding. I played along and said “yes,” as to not embarrass him, but because I was on antibiotics for my sepsis, he had to call the doctor to ensure this was ok. He had NO indication I had an abortion.

Negative experiences with the Military Health System not only impacted participants during their abortion process but also in their subsequent interactions with military healthcare professionals.

I am currently pregnant and I felt like I was judged for an elective termination of [my] first pregnancy when first starting my prenatal care; I also have great anxiety every time I see my PCM [Primary Care Manager] and they have you fill out the intake questionnaire where it asks if you are pregnant or previously been pregnant and how many live births.

## 4 Discussion

This study explored the lived experiences of servicewomen who had an abortion while serving on active duty in the US military. While previous qualitative studies have examined servicewomen seeking abortion care, they had smaller sample sizes, primarily focused on systemic barriers, and lacked the application of a comprehensive conceptual framework focused on abortion access. The use of Coast et al.'s (2018) framework enabled a more nuanced exploration of the lived experiences of ADSW in terms of their personal characteristics that influenced their decision to seek abortion care, their actions to obtain abortion care and the influence of both immediate environments and broader military legal and healthcare systems. By applying a framework that integrates both individual and systems‐level factors, this study offers a more complete understanding of how ADSW perceive and navigating abortion care within the context of active‐duty military service. Notably, we found that some ADSW who received medical management for miscarriage self‐identify as having had an abortion or termination despite the absence of fetal cardiac activity. Additional insights include the experiences of servicewomen seeking care due to fetal anomalies, and those who accessed TRICARE covered abortion care in MTFs.

The majority of participants (62%) sought abortion care due to an unplanned pregnancy, which is comparable to earlier studies that found 66%–78% of ADSW sought abortion care due to unplanned pregnancies [19, 20]. ADSW who had what they considered to be “medically necessary” abortions often felt that they should be treated differently from those who had elective abortions. Notably, 10% described what they considered to be abortion‐related experiences related to miscarriage or ectopic pregnancy management, indicating a lack of knowledge. Although medical miscarriage management in the first trimester aligns with abortion care procedures, it does not involve terminating the heartbeat of the fetus—a distinction that some participants did not fully understand. This lack of understanding contributed to subsequent emotional distress and mental health concerns in a population already at risk for poor mental health outcomes [30]. To our knowledge, this study is the first to identify this deficit in knowledge among servicewomen receiving care for miscarriages.

Several themes highlighted how current policies undermine mission readiness for ADSW seeking abortion access. Pregnancy carries higher health risks than abortion and requires a longer recovery time [31], yet ADSW were obligated to pay for abortions for catastrophic fetal anomalies or seek care at substandard facilities which led to long‐term health consequences. Previous studies have also described women receiving suboptimal abortion care and insufficient recovery times due to restrictive TRICARE policies or military leadership requiring personnel to perform specific shifts or duties [10, 20]. Such practices, which prevent servicewomen from adhering to evidence‐based guidelines for care and recovery, pose significant risks to the health and well‐being of servicewomen. Furthermore, the lack of support for ADSW at MTF for both abortion counseling and receiving abortion services may foster medical mistrust and undermine the potential for future therapeutic patient‐provider relationships, potentially decreasing the likelihood that ADSW will seek care or communicate openly with providers.

Most ADSW were concerned about medical reporting systems that compromised personal privacy due to the stigmatized nature of abortion in the military. The majority did not want their leadership or peers to know about their decision to seek abortion care, which is consistent with previous studies [19]. However, some were forced to disclose their decision to access care due to the leave and pass policies. At times, this disclosure led to personal and professional repercussions from leadership that did not agree with the ADSW's decision to terminate the pregnancy. These findings reinforce the importance of the DOD's 2022 decision to extend mandatory reporting of pregnancy until 20‐weeks' gestation, allowing patients more time to make private healthcare decisions.

However, despite progress to protect patient privacy, abortion care access persists as a notable area of health disparity in the Military Health System. Although abortion care is not classified by the DOD as an “elective procedure,” it is the only nonelective procedure in which servicemembers are expected to arrange their own care with little or no support from military healthcare professionals, to include finding a provider, means of transportation, and payment [12, 14]. Furthermore, while pregnancy options counseling is highly subjective across MTFs and individual military healthcare providers with no standardized procedures or guidance to ensure high‐quality, evidence‐based care, other health concerns (ranging from deployment health to perinatal care) do have DOD‐wide procedural instructions for standardized care [32, 33].

### 4.1 Limitations

This study has several limitations. Demographic data were not collected to ensure participant privacy and facilitate honesty, which may affect the generalizability of the findings. The self‐reported nature of the data introduces the possibility of inaccuracies, as some participants may have sought abortion care, but did not disclose it, leading to their omission from the analysis. Additionally, the use of convenience sampling may have introduced participant bias. Data collection took place prior to DOD policy changes in 2022 delaying notification of pregnancy to leadership to 20‐weeks' gestation age, limiting the transferability of the results to ADSW currently seeking abortion care. Despite these limitations, the sample (*N* = 178) represents a robust, rich data set from a population that is often difficult to access. The decision to use Coast et al.'s (2018) framework to create a priori codes could have resulted in potential themes being missed, however, we found it to be a good fit for the research objectives as it focused on the individual experience as opposed to systemic barriers.

#### 4.1.1 Research and Policy Recommendations

Future research should explore how systemic barriers to abortion care impact the overall health and mission readiness of ADSW who were able and unable to obtain abortion care. The knowledge, attitudes and beliefs of military healthcare providers regarding abortion care, and willingness and aptitude for pregnancy options counseling (i.e., carry to term, adoption or termination), should be examined as many participants felt judged by providers or were not offered options counseling. This would allow the DOD to develop training and education materials for providers and patients. Studies exploring the effectiveness of recent DOD policy changes regarding access to abortion care (e.g., delayed notification of pregnancy to leadership) should be performed to assess if they have achieved their intended effect.

The DOD should continue to develop and implement policies to protect the medical privacy of ADSW who require abortion care. DOD‐wide guidance regarding pregnancy options counseling that reinforces the authority of the provider to educate patients and standardizes options and resources should be adopted immediately. The DOD should also create a streamlined referral process for pregnancy counseling should military providers refuse such counseling to ADSW on the grounds of personal religious beliefs. Such a program could be centralized and utilize telehealth to increase accessibility. These measures would reduce the barriers to accessing abortion care for many ADSW. Furthermore, the DOD should permit ADSW to pay for noncovered abortions at the MTF in countries or states with full or partial abortion bans to reduce the likelihood of delayed or substandard care. Military healthcare providers should receive mandatory education on TRICARE coverage of abortion care and non‐TRICARE alternatives (e.g., abortion funds) to facilitate access to care and ensure the best possible outcomes and mission readiness of ADSW. These measures would address ongoing systemic barriers and work to improve access to effective and equitable abortion care for all servicewomen.

## Conflicts of Interest

The authors declare no conflicts of interest.

## Supporting information


**Data S1:** psrh70064‐sup‐0001‐supinfo.docx.

**Data S2:** psrh70064‐sup‐0002‐supinfo.docx.

**Data S3:** psrh70064‐sup‐0003‐supinfo.docx.

## Data Availability

Research data are not shared.
