# Supplementary material for: “It's My Dark Secret”: A Qualitative Study on the Abortion Experiences of US Active‐Duty Servicewomen
Source: Perspect Sex Reprod Health. 2026 Apr 29;58(2):159–69. doi: 10.1111/psrh.70064 (PMC13247853; doi:10.1111/psrh.70064)
Supplement: Supplementary file 2 — Data S2: psrh70064‐sup‐0002‐supinfo.docx. [file PSRH-58-159-s002.docx]

**Supplement B. Study Questionnaire Response Rates and Characteristics**

| **Question** | **Response Rates** | **Response Lengths** |
| --- | --- | --- |
| 1. Do you believe Tricare covers adequate reproductive care for females? (e.g., birth control, infertility treatments, abortion access, OB care, etc.) If you answered no to question #1, please specify: | 142/178 (83%) | Range: 2 – 312 words  Average: 29 words |
| 6. Did you take personal leave leading up to, to obtain, or following the termination? If you answered yes to question 6, please specify each leave and intent (e.g. to travel, to heal, to grieve): | 88/178 (49%) | Range: 1 – 110 words  Average: 10 words |
| 7. Did you have to travel more than one hour to have access to health care associated with pregnancy termination? If you answered yes to question 7, please specify: | 69/178 (39%) | Range: 1 – 66 words  Average: 10 words |
| 8. How much in out-of-pocket expenses did you incur as a result of travel and termination access? | 161/178 (90%) | Range: 1 – 39 words  Average: 6 words |
| 14. Do you believe you’ve experienced discrimination because of a termination or others’ belief you terminated a pregnancy? If you answered yes to question 14, please specify: | 58/178 (33%) | Range: 3 – 248 words  Average: 34 words |
| 23. Is there anything else you would like to share about your experience accessing birth control or abortion care? If so, please free-text in the below box: | 73/178 (41%) | Range: 1 - 334  Average: 58 words |
