# Supplementary material for: “It's My Dark Secret”: A Qualitative Study on the Abortion Experiences of US Active‐Duty Servicewomen
Source: Perspect Sex Reprod Health. 2026 Apr 29;58(2):159–69. doi: 10.1111/psrh.70064 (PMC13247853; doi:10.1111/psrh.70064)
Supplement: Supplementary file 3 — Data S3: psrh70064‐sup‐0003‐supinfo.docx. [file PSRH-58-159-s003.docx]

**Supplement C. Research Team Reflexivity Statement**

Research Team Reflexivity: We are a multi-ethnic group of American women. Six of the eight authors are currently serving or have previously served in the United States military (Army and Air Force) on active-duty status as either officers or enlisted personnel. All but one are combat veterans. These authors identify as insider researchers who have similar lived experiences to the questionnaire respondents regarding access to reproductive healthcare in a military setting. Five of the authors are healthcare professionals, three of whom are currently serving in the military. Members of the research team included specialists in policy, women’s health, and military health care delivery.

Interpersonal Reflexivity: The community-engaged research approach towards data collection facilitated respondent trust and buy-in. The research questions and questionnaire were developed by active-duty servicewomen meaning that respondents saw their unaddressed reproductive health concerns addressed in a nuanced and military-culturally appropriate manner. The decision to not require the use of a military identification card for verification and permitting respondents to remain completely anonymous facilitated greater levels of honesty. Respondents commented very favorably upon the community-engaged research aspect of data collection.

Methodology Reflexivity: For data analysis, we deliberately chose a framework that centers the experiences of individuals along the continuum of abortion care access. This approach allowed us to focus on the lived experiences of respondents within the context of military service, instead of exclusively identifying system-level factors that drive access to abortion care. Those with military service met frequently to discuss modifications to the Coast et al. framework and the initial codebook development. After these meetings, this material was assessed by the non-military members of the research team. We believe that having research team members with military experience code the data allows for a richer interpretation and analysis due to shared lived experiences as members of a sub-culture. Collaborating with non-military coders further helped reduce bias. Examination of personal bias consisted of memos and open discussion during consensus meetings and theme development of how personal experience could introduce bias.

Contextual Reflexivity: Respondents were concerned with their anonymity, and it is possible that, although no personal data was collected, their replies omitted information due to fear of identification and subsequent judgement or professional repercussions. This data was collected prior to the Dobbs Decision in 2022 at a time when many servicewomen were concerned about reproductive access. Data analysis was performed in summer and early autumn of 2024, after the Department of Defense adopted policies meant to facilitate abortion access for service women. These policies were repealed in January 2025. Organizations meant to advocate for servicewomen (e.g., Women’s Initiative Teams, SWORD Athena) have been disbanded under the current administration.
